# High-Concentration Capsaicin Patch and Oral Pregabalin as Second-Line Therapy for Intercostobrachial Neuralgia After Breast Cancer Surgery: Open-Label Follow-Up of a Multicenter Randomized Controlled Clinical Trial

**DOI:** 10.3390/cancers18142355

**Published:** 2026-07-21

**Authors:** Denis Dupoiron, Florent Bienfait, Valérie Seegers, Sabrina Jubier-Hamon

**Affiliations:** 1 Département d’Oncoréhabilitation, Institut de Cancérologie de l’Ouest, 49055 Angers, France; florent.bienfait@ico.unicancer.fr (F.B.); sabrina.jubier-hamon@ico.unicancer.fr (S.J.-H.); 2 Biometrics Department, Institut de Cancérologie de l’Ouest, 49055 Angers, France; valerie.seegers@ico.unicancer.fr

**Keywords:** breast neoplasms, capsaicin, efficacy, mastectomy, neuropathic pain, patient preference, pregabalin, safety

## Abstract

Background: The CAPTRANE trial evaluated the efficacy of randomly assigned high-concentration capsaicin patch (HCCP) versus daily oral pregabalin (PGB) in adults with chronic neuropathic pain (DN4 ≥ 4) post-breast cancer surgery. The objective of its 4-month open-label extension following patient’s self-selection of their second treatment (HCCP, PGB, or none) was to explore efficacy and safety of various strategies. Methods: The study was conducted in France between March 2019 and November 2022. At each clinic visit, we assessed pain intensity (NRS, 0–10), painful area (cm^2^), mood (HADS), and quality of life (EQ-5D-5L). Standard statistical tests were used. Results: Data from 116 patients (all females; 76% aged < 65 years) were collected. At Month 2, 71% (46/65) of HCCP-treated patients received a second HCCP application and none switched to PGB, whereas 27% (14/51) continued on PGB and 49% (25/51) of PGB-treated patients switched to HCCP. At Month 6, NRS scores had decreased in HCCP-treated patients, including PGB/HCCP-treated patients, with a reduction of −2.0 [−4.0; 0.0] and −3.0 [−4.0; −2.0] in the HCCP/HCCP and PGB/HCCP group, respectively (median[interquartile]). Adverse events aligned with those expected with HCCP and PGB; none were serious. Conclusions: This first study to examine switching between PGB and HCCP treatments for chronic intercostobrachial neuropathic pain after breast cancer surgery showed patients’ preference for HCCP, confirmed the benefit of repeated HCCP applications, and indicated that HCCP could be preceded by PGB without compromising efficacy or safety. These findings suggest that HCCP may be used early in this population, and PGB initiated before HCCP to relieve patients waiting for HCCP application.

## Simple Summary

CAPTRANE, a randomized controlled trial conducted in France (2019–2022), compared high-concentration capsaicin patch (HCCP) with oral pregabalin (PGB) two months after randomization in patients with intercostobrachial chronic neuropathic pain after breast cancer surgery. The subsequent 4-month open-label extension period that included 116 patients who chose their subsequent treatment (HCCP, PGB, or none) is reported here. Outcomes include pain intensity, painful area, mood, and quality of life. More than 2 out of 3 patients initially treated with HCCP chose to continue with HCCP, while less than 1/3 of those on PGB continued PGB. By Month 6, pain scores decreased in all patients treated with HCCP, including those who switched from PGB to HCCP. Tolerability was consistent with the known safety profile of HCCP and PBG. Overall, results indicate a preference for HCCP, support repeated HCCP applications, and suggest that PGB may be used as a temporary option for patients waiting for HCCP application.

## 1. Introduction

The International Association for the Study of Pain (IASP; https://www.iasp-pain.org) defines chronic pain after breast surgery (CPBS) as pain that develops or increases in intensity following a surgical procedure in the breast area and that persists beyond the healing process (i.e., at least 3 months post-surgery) [1]. CPBS is predominantly neuropathic, manifesting as pain localized to the surgical site, projected to the territory innervated by affected nerves, and/or referred to a dermatome (https://www.iasp-pain.org). In clinical practice, validated instruments such as the *Douleur Neuropathique en 4 Questions* (DN4) can help in screening for neuropathic signs and symptoms [2].

CPBS is recognized as a therapy-related complication [3,4]. It is frequent within the year after breast cancer surgery. Globally, breast cancer is the most common cancer among women, with 2.3 million new cases and 670,000 deaths in 2022 [5]. Whereas annual rates of new cases continued to increase in most countries, mortality rates tended to decrease in countries with a high Human Development Index [5], leading to a greater number of survivors. A meta-analysis on the prevalence of pain in cancer survivors with a solid tumor suggested that about 50% of survivors report pain more than 3 months after completing curative treatment [6]. In 2021, in France, where breast cancer survival at five years was estimated at 88%, 10–24% of women reported persistent moderate to severe neuropathic pain following surgery, highlighting the importance of early detection and targeted interventions (https://www.cancer.fr). CPBS, especially when a neuropathic component is present, often leads to long-term disability and reduced quality of life [7,8]. Furthermore, it is well-established that pain and depression are linked by a bidirectional relationship: they frequently coexist, respond to similar treatments, exacerbate one another, and share biological pathways and neurotransmitters [9].

Pharmacological treatment options for neuropathic CBPS include antidepressants (e.g., duloxetine, tricyclic antidepressants) and anticonvulsants (e.g., pregabalin) [10]. Pregabalin, an alpha-2-delta ligand, is internationally recommended as first-line in neuropathic pain [11]. It is commonly prescribed to prevent or relieve CPBS, based on the results of studies demonstrating reductions in postsurgical neuropathic pain intensity, particularly when pregabalin was initiated perioperatively [12,13]. Despite the presence of specialized pain and oncology centers, adult patients with CPBS often remain untreated, partly due to the inconsistent benefit-to-risk ratio of available pharmacologic treatments [6,14]. Moreover, the selection of the therapeutic options is influenced by concomitant medications, particularly in women with breast cancer, as adverse events may overlap with those of oncologic treatments such as aromatase inhibitors [15,16]. In this context, topical therapies may be preferred as they reduce the risk of systemic adverse events. In France, the 8% capsaicin patch is approved for all peripheral neuropathic pain indications. In contrast, the 5% lidocaine plaster is approved only for the treatment of postherpetic neuralgia.

The high-concentration capsaicin (179 mg) patch (HCCP) (QUTENZA^®^; Grünenthal, Aachen, Germany) has been authorized for use in Europe since 2009. It offers an alternative strategy for managing localized postoperative neuropathic pain (https://www.ema.europa.eu). It is indicated for the treatment of peripheral neuropathic pain in adults and may be used as a standalone treatment or in conjunction with oral analgesics [17,18]. Its safety profile is predominantly characterized by transient, localized application-site reactions. A case report and a clinical study have demonstrated its effectiveness in CPBS [19,20]. However, to the best of our knowledge, its efficacy has never been explored comparatively to pregabalin to relieve CPBS before the CAPTRANE study.

CAPTRANE (NCT03794388) stands for “Early CAPsaicin: evaluation in the TReAtment of post breast surgery intercostobrachial NEuralgias”. It is a two-period clinical study based on the method of attribution of the pain treatment: randomization (Period 1) or patients’ choice (Period 2). Results of Period 1, a phase 3, multicenter, open-label, randomized controlled trial, have been published by Dupoiron et al. [21]. They showed the noninferiority at 2 months (M2) of early HCCP administration compared to oral PGB in adults with chronic intercostobrachial neuropathic pain following primary breast cancer surgery. The second period was an open-label follow-up aimed at evaluating the effectiveness and safety at 6 months (M6) irrespective of treatment assignment at randomization. Indeed, at M2, patients could discontinue treatment, continue their treatment, or switch between treatments. This article presents the results of this open-label extension.

## 2. Materials and Methods

### 2.1. Study Design

**Figure 1:**
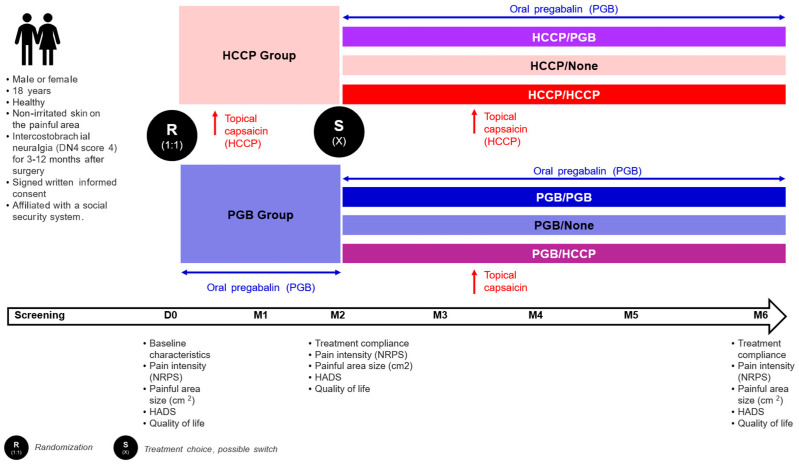
Study design. D: Day; HADS: Hospital Anxiety-Depression Scale; HCCP: High Concentration Capsaicin Patch; M: Month; PGB: Pregabalin; NPRS: Numeric Pain Rating Scale.

The study design is presented in Figure 1. On day 0 (D0), each participant was randomly assigned in a 1:1 ratio to receive either topical HCCP or oral pregabalin (PGB). At M2, participants assigned to the HCCP group were offered the options to stop treatment, to receive a second application of HCCP (to be administered at least one month later), or to switch to oral PGB. Those assigned to the PGB group were offered the options to stop treatment, to continue PGB, or to switch to receive a first application of HCCP. The continuation of study treatment depended on the tolerability of the treatment and the persistence of neuropathic pain. Outcome measures were collected on D0 and at M2 and M6.

### 2.2. Participants and Settings

Recruitment started in March 2019 and was stopped in May 2022. Participants were enrolled and randomized across nine expert pain centers in France (Angers, Villejuif, La Roche-sur-Yon, Lille, Lyon, Saint-Herblain, Saint-Cloud, Montpellier, and Toulouse).

Eligible participants were male and female adult patients aged 18 years or older who had undergone primary surgical resection for breast cancer. Specific inclusion criteria required patients to have intact, non-irritated skin over the areas of neuropathic pain, and to report intercostobrachial neuropathic pain confirmed by a DN4 score equal to or greater than 4 within 3 to 12 months following surgery. Participants were required to be affiliated with the French social security system. Patients with prior exposure to HCCP or PGB after surgery but before inclusion, patients under opioid treatment (>80 mg/day), patients with diabetes, and patients with specific contraindications to at least one of the two treatments were not included in the study to avoid confounding factors potentially impacting the interpretation of data (for further details, see Supplementary Table S1).

### 2.3. Randomization and Blinding

On D0, randomization was executed using a minimization algorithm without random allocation components and was implemented through the Ennov Clinical software (V10.3) system. Allocation was stratified by participating center, age group (≤65 or >65 years), and baseline pain intensity (Numeric Rating Scale, NRS score < 5 or ≥5) and anxiety status (Hospital Anxiety-Depression Scale scores for anxiety, HADS-A score < 8, 8–10, >10 [22]) assessed before randomization. Due to the nature of the treatments and their route of administration, including HCCP administration constraints, the first study period was conducted as an open-label trial. Standardized data collection procedures and central data monitoring were used to reduce assessment bias. Pain surface maps were centrally assessed by SJH, who was blinded to group allocation and trial treatment received.

### 2.4. HCCP and PGB Treatments

For patients assigned to HCCP treatment and at each visit when HCCP was applied, patch application details, including the number of patches and date of use, were recorded. During each treatment session, a maximum of two patches were applied to the painful area for a maximum of 60 min. If more than two patches were required, a second session could be performed seven days later to complete the application. These two sessions were considered one single administration. In case of pain persistence or recurrence, HCCP administration was repeated (if the patients agreed) with a minimum of three months between the first and second administration. The same procedure as described above was implemented for patients who switched from PGB to HCCP treatment. Overall, patients could receive up to two administrations of HCCP.

Patients included in the PGB group (or switching from HCCP to PGB treatments at M2) were treated with 50 mg/day of PGB, divided into two doses (or 25 mg/day if deemed appropriate by the treating physician) and up-titrated every 3–7 days with up to 50 mg/day to a maximum dose of 600 mg/day, as appropriate. PGB adherence was tracked through case report forms specifying maximum dose reached and whether the patient maintained ≥75% compliance [21].

### 2.5. Outcome Measures

Pain intensity was assessed using an 11-point numeric rating scale (NRS) ranging from 0 (no pain) to 10 (worst pain imaginable). Patients rated the maximal pain intensity (MPI) experienced during the previous 24 h. Response to treatment was defined as either a decrease of at least 20% in the NRS score compared to baseline (D0) or a post-treatment score of less than 3.

The size of the painful area (cm^2^) was assessed through visual pain mapping, followed by centralized reading and quantification of standardized anatomical diagrams.

Quality of life was assessed by the French-language version of the EQ-5D-5L (https://euroqol.org). The EuroQol 5-dimension, 5-level (EQ-5D-5L) questionnaire comprises a visual analog scale (VAS) by which patients rate their health, as well as a descriptive system with five dimensions (mobility, self-care, usual activities, pain/discomfort, and anxiety/depression) [23]. Its score is calculated from the combination of responses of the five dimensions and ranges from 0 (death) to 1 (full health). This tool is frequently used in breast cancer, including after surgery [24,25].

Anxiety and depression were assessed using the HADS [26]. This scale includes 14 items split into anxiety and depression subscales (HADS-A and HADS-D). Used thresholds were the following: 0–7 (normal), 8–10 (borderline), and >10 (anxiety/depression).

All instruments were completed at standardized time points: D0, M2, and M6, with administration performed in clinical settings using paper-based case report forms and subsequently digitized.

Adverse events reported during the whole study period were classified using the Common Terminology Criteria for Adverse Events (CTCAE) version 5.0. Only adverse events with a grade of 2 or higher were collected. Grade 2 adverse events were considered moderate, and grade 3–5 adverse events were considered severe.

### 2.6. Statistical Analysis

The primary objective of the CAPTRANE study was to demonstrate the non-inferiority of HCCP treatment compared to PGB treatment on pain intensity (NRS) at M2. The results of this randomized Period 1 have been published elsewhere [21]. Here, we present results at M6 in all patients who received at least one of the two treatments (at least one dose for PGB) according to the treatment they actually received. No sample size calculation was conducted for Period 2. We excluded from the analysis all patients who did not receive either of the two study treatments. At M6, groups were defined according to the actual treatment exposure during Periods 1 and 2, yielding six distinct categories: HCCP/None, HCCP/HCCP, and HCCP/PGB for patients who received HCCP in Period 1 followed respectively by no treatment, HCCP, or PGB in Period 2; PGB/None, PGB/PGB, PGB/HCCP, and for those who received PGB in Period 1 followed respectively by no treatment, PGB, or HCCP in Period 2. An additional group corresponding to patients receiving both PGB and HCCP during Period 1 (considered a protocol deviation) and HCCP during Period 2 (PGB + HCCP/HCCP) was also defined but will not be discussed given the small sample size (*n* = 6).

Statistical analyses were conducted based on the type of variables. Numeric and continuous variables were summarized using means, standard deviations (SDs), medians, and interquartile ranges (IQRs). Between-group comparisons of NRS pain scores and repeated measures outcomes were assessed using ANOVA models. Paired changes over time were analyzed using Student’s *t*-test or the Friedman test, or Wilcoxon signed-rank tests for non-parametric distributions. No imputation of missing data was performed for these follow-up analyses. All statistical analyses were performed using R (R Foundation for Statistical Computing, Vienna, Austria. <https://www.R-project.org/>) version 4.1.2. A threshold of *p* < 0.05 was used to define statistical significance.

### 2.7. Ethical Considerations

The protocol received approval by the appropriate institutional review board/independent ethics committee at each participating study site and by any relevant competent authority (Comité de Protection des Personnes Sud Méditerranée IV, CPP: 18 11 04). All participants provided written informed consent prior to enrollment. The study (NCT03794388) was conducted in accordance with the protocol and ethical principles within the Declaration of Helsinki, and in accordance with the Council for International Organizations of Medical Sciences International Ethical Guidelines and the International Conference on Harmonization Good Clinical Practice Guideline.

## 3. Results

### 3.1. Participants

**Figure 2:**
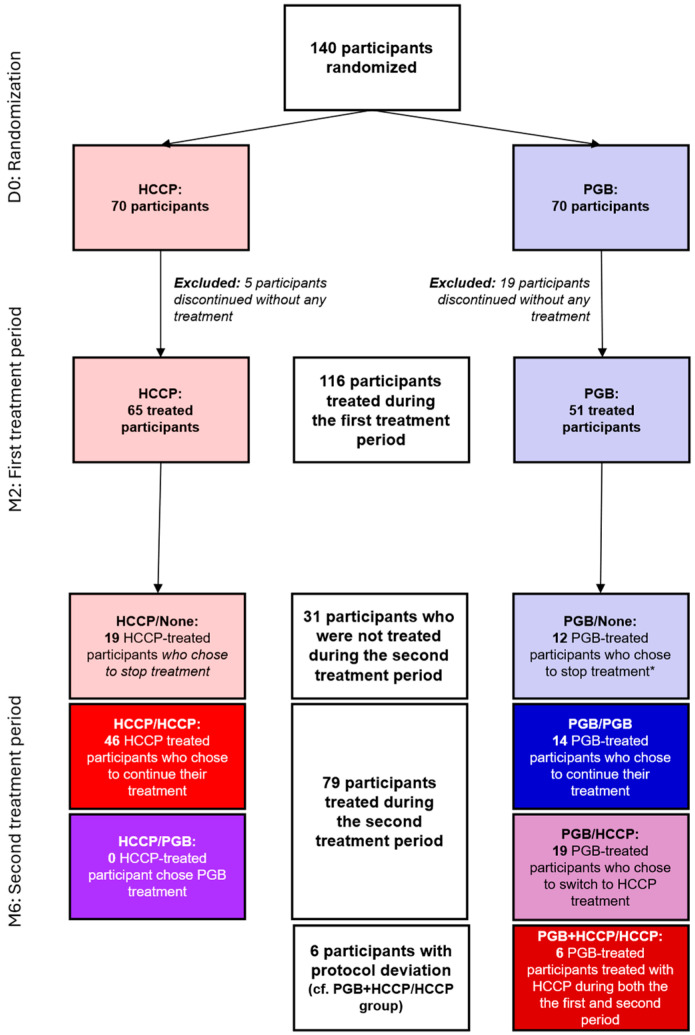
Study flow chart. D, day; HCCP, high-concentration capsaicin (179 mg) patch; M, month; PGB, pregabalin. * Among the 12 patients from the PGB/None group, 7 patients who initially chose to continue treatment ultimately did not receive it.

A total of 140 patients were randomly assigned to receive HCCP (*n* = 70) or PGB (*n* = 70) treatment. Of these, 24 patients (5 and 19, respectively) withdrew from the study prior to any treatment administration. The 116 remaining patients (65 and 51, respectively) constituted the study population. Of them, 85 were treated during the second period: 71 with HCCP (HCCP/HCCP, 46; PGB/HCCP, 25) and 14 with PGB (PGB/PGB) (Figure 2).

All the patients were female, predominantly under the age of 65 years (76%, *n* = 88); 28% (*n* = 33) underwent a mastectomy and 34% (*n* = 40) axillary node dissection. Lymphedema (22%, *n* = 26) was the most frequently reported post-operative complication. Most patients were under radiotherapy (81%, *n* = 94) and 70% (*n* = 81) took hormonal therapy. HADS-A and HADS-D scores consistent with clinically significant symptoms (≥11) were observed in 27% (*n* = 31) and 15% (*n* = 17) of patients (Table 1).

### 3.2. Treatment Administration and Switching

The mean (SD) number of patches administered to each patient assigned to the HCCP group was 1.3 (0.7) during the first period (*n* = 65) and 1.3 (0.6) during the second period (*n* = 46), and 1.2 (0.5) for those assigned to the PGB group who switched to HCCP (Table 2).

PGB compliance was good, with 94% (48/51) of patients assigned to PGB treatment taking ≥ 75% of prescribed doses during the first period and 100% (14/14) for those continuing PGB treatment (Table 2).

Among patients treated with HCCP during Period 1, 71% (46/65) had a second application (HCCP/HCCP); no patients chose to switch to PGB. Among patients treated with PGB during Period 1, 27% (14/51) continued PGB treatment (PGB/PGB), 53% (27/51) opted to switch to HCCP, and 49% (25/51) received HCCP (PGB/HCCP) (Figure 2).

In the HCCP group, 47% (9/19) of patients did not get a second HCCP treatment due to pain relief, and 10% (2/19) because of poor tolerability. In the PGB group, 48% (7/12) of patients chose to continue treatment with either PGB (5/12) or HCCP (2/12) but were not treated.

### 3.3. Maximal Pain Intensity and Treatment Response

In the total observed population, the maximal pain intensity (MPI), which was 6.0 [5.0; 7.0] at baseline (median [IQR] NRS score), decreased to 5.0 [2.0; 6.0] at M2 and 4.0 [2.0; 6.0] at M6, indicating significant pain reduction over time (Friedman test, *p* < 0.01).

At baseline, MPI did not differ significantly across treatment groups (Kruskal–Wallis test, *p* = 0.548), with median values ranging from 5.0 [4.5; 6.0] in the HCCP/None group to 7.0 [6.0; 8.0] in the PGB/HCCP group (Supplementary Table S2). A significant between-group difference was observed at M2 (*p* = 0.002), with the lowest scores for the HCCP/None (0 [0; 4.0]) and PGB/None (2.5 [0; 4.5]) groups, but not at M6 (*p* = 0.430). At M6, median scores [IQR] ranged between 2.0 [0; 4.5] in the HCCP/None group and 5.5 [3.0; 7.0] in the PGB/PGB group.

At M2, median MPI scores had decreased across all groups, with the largest reduction observed in the HCCP/None group (−4.0 [−5.0; −2.0]). Early response to treatment varied between groups, although pain intensity tended to converge over time. Median changes from baseline to M6 did not differ significantly between treatment groups (*p* = 0.410), and ranged between −3.5 [−4.2;−1.8] in the HCCP/None group and −1.0 [−2.8; 0] in the PGB/PGB group, while, in contrast, median changes from D0 to M2 and from M2 to M6 differed significantly between groups (*p* = 0.016 and *p* = 0.010, respectively).

**Figure 3:**
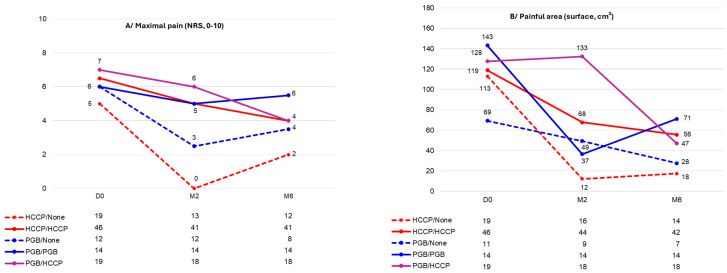
Median maximal pain intensity (NRS, 0–10) (**A**) and median painful area (surface, cm^2^) (**B**) from baseline to month 6 by treatment group (rounded median values). HCCP, high-concentration capsaicin (179 mg) patch; NONE, no treatment; NRS, numeric rating scale (0–10); PGB, Pregabalin. D0, baseline measurement (day 0); M2, measurement at month 2; M6, measurement at month 6.

As shown in Figure 3A, median MPI scores decreased markedly after treatment in the HCCP/None and PGB/None groups. In contrast, pain reduction was more gradual but consistent in the HCCP/HCCP and PGB/HCCP groups. Pain intensity remained stable between M2 and M6 in PGB-treated patients who chose to continue PGB treatment.

At M6, 77% (89/116) of patients were classified as responders (defined as a ≥20% decrease in MPI score from baseline or an MPI score < 3). All other patients were classified as non-responders (33.7%, 27/116), including 18/116 patients (16%) without data. The percentage of missing data was higher in the HCCP/None (37%, 7/19) and PGB/None (33%, 4/12) than in the other groups, e.g., HCCP/HCCP (11%, 5/46), PGB/PGB (0%, 0/12), and PGB/HCCP (5%, 1/19) groups. Responder rates were 63% (12/19) in the HCCP/None group and 67% (8/12) in the PGB/None group and went up to 100% (12/12 and 8/8, respectively) if missing data were excluded. In the HCCP/HCCP, PGB/PGB, and PGB/HCCP groups, responder rates were 78% (36/46), 86% (12/14), and 89% (17/19) but 88% (36/41), 86% (12/14), and 94% (17/18) after exclusion of missing data in the corresponding groups.

### 3.4. Painful Area

In the total observed population, the surface of the painful area (cm^2^), which was 118.9 [55.5; 180.4] cm^2^ at baseline (median [IQR]), decreased to 59.7 [31.6; 123.5] cm^2^ at M2 and to 46.7 [16.5; 89.1] cm^2^ at M6, indicating significant improvement in the whole study population (Friedman test *p* < 0.0001).

At baseline, the surface of the painful area (cm^2^) did not differ significantly across treatment groups (Kruskal–Wallis test, *p* = 0.542), with median values ranging from 69.3 [42.3; 174.8] in the PGB/None group to 143.2 [84.7; 173.0] in the PGB/PGB group (Supplementary Table S3).

At M2, the median surface had decreased across all groups, with the lowest surface in the HCCP/None group (12.2 [4.6; 41.2]). A significant difference was observed between groups at M2 (*p* = 0.0001) but not at M6 (*p* = 0.120). At M6, the lowest surface was observed in the HCCP/None group (17.5 [1.0; 46.5]) and the highest in the PGB/PGB group (71.2 [32.6; 126.0]). Changes from baseline to M2 and M6 did not differ significantly between treatment groups (*p* = 0.457 and *p* = 0.512, respectively). In contrast, changes from M2 to M6 differed significantly between treatment groups (*p* = 0.004), with the largest decrease being observed in the PGB/HCCP group (−84.8 [−113.0;−35.1] and an increase in the PGB/PGB group (13.7 [−10.9; 41.0]).

As shown in Figure 3B, the reduction in the median painful surface was marked after treatment in the HCCP/None group. In contrast, it was more gradual but consistent in the HCCP/HCCP group. After the second treatment, painful area markedly decreased in the PGB/HCCP group but not in the PGB/PGB group.

### 3.5. Quality of Life, Anxiety, and Depression

In the total observed population, the EQ-5D-5L utility score (median [IQR]) increased from 0.642 [0.487; 0.798] at baseline to 0.821 [0.354; 0.932] at M2 and 0.798 [0.564; 0.867] at M6, indicating significant improvement (Friedman, *p* = 0.002). No statistically significant differences in evolution were observed according to the treatment received, indicating similar improvement in all treatment groups (See Supplementary Table S4).

At baseline, 30.7% of the patients had definite symptoms of anxiety (HADS-A score > 10), with no statistically significant difference between groups (*p* = 0.769). From baseline (D0) to M6, no statistically significant differences in the distribution of patients between groups were observed across normal, borderline, and depressive symptom categories and across time (*p* > 0.05). Similarly, the percentage of patients with definite symptoms of depression (HADS-D score > 10), which was 15.5% at baseline and similar in all groups (*p* = 0.894), remained stable in all treatment groups over time (*p* > 0.05) (Supplementary Table S5).

### 3.6. Safety

No serious adverse event was reported during the study. A total of 61 adverse events of moderate or severe intensity probably related to HCCP or PGB administration were reported. These adverse events were reported by a total of 38 patients (33%), 12 randomized to receive HCCP and 26 randomized to receive PGB; they aligned with those expected with HCCP and PGB. Most of them (*n* = 41) occurred during period 1 (i.e., from D0 to M2, randomized period). Of these 61 adverse events, 6 were considered severe (grade 3).

In patients randomized to HCCP (HCCP/none or HCCP/HCCP groups), the most frequently reported adverse events of moderate to severe intensity probably related to HCCP administration were application site burning (*n* = 9) and pain (*n* = 5). Of the nine cases of burning at application site, two were of severe intensity (one patient reported excruciating pain) and seven were of moderate intensity (one patient reported a sensation of warmth). In patients from the PGB/None and PGB/PGB groups, the most frequently reported adverse events of moderate to severe intensity probably related to PGB administration were vertigo (*n* = 4) and somnolence (*n* = 3). In patients from the PGB/HCCP and PGB + HCCP/HCCP, the most frequently reported adverse events during the trial were somnolence (*n* = 6), vertigo (*n* = 5), headache (*n* = 4) and application site burning (*n* = 4).

## 4. Discussion

The primary finding of this longitudinal analysis was that early treatment with HCCP is effective in patients with peripheral neuropathic pain following breast surgery. A single HCCP application resulted in a reduction in pain intensity and in the surface of the painful area at M2, which was sustained at 6 months in 14% (9/65) of patients without further HCCP treatment. These findings align well with those of the QUEPP study [27], which demonstrated that patients treated early (i.e., within 6 months) after diagnosis of postsurgical neuropathic pain experienced the best treatment outcomes, with 61.7% of patients achieving a 30% reduction in baseline pain intensity after 12 weeks of HCCP treatment. Overall, some patients (19/65) decided to stop treatment as their median pain levels were at 0 after a single treatment. At M6, the high number of missing data in the HCCP/None group may have led to an underestimation of the proportion of responders in this group. The high amount of missing data may be due to patients experiencing substantial clinical improvement decreasing their willingness to return for follow-up assessments. Owing to the low sample size and high level of missing data, it was not possible to identify potential factors contributing to early treatment response.

Repeated HCCP treatment in 46/65 of those originally assigned to HCCP treatment led to a further decrease in MPI and the surface of the painful area. These findings align with those from CASPAR, a retrospective registry study in patients with neuropathic pain due to peripheral nerve injury following trauma or surgery that demonstrated increasing benefit with repeated treatments [28]. Given the small sample size in the PGB groups, meaningful conclusions regarding changes in MPI scores or the size of the painful area over time are limited.

Furthermore, this longitudinal analysis showed that switching from PGB to HCCP treatment at M2 allowed pain intensity or painful area to further decrease, and at M6 median MPI scores were similar in patients initiated on PGB and then switched to HCCP as in those assigned to HCCP and continuing HCCP. The painful area reduction was also similar at M6 in those who continued HCCP after a first treatment as compared to patients initially treated with PGB. This is informative for clinical practice as, due to practical constraints, HCCP treatment may not be readily available at the time of diagnosis. Postponing HCCP treatment while awaiting staff availability and temporarily using oral therapies to bridge the gap between the diagnosis of neuropathic pain and HCCP treatment may assist patients in managing their pain without negatively impacting the long-term outcomes of future HCCP treatment.

In addition, in this study where patients could discontinue treatment, receive a second HCCP application, or switch to oral PGB, patients demonstrated a preference for topical therapy over oral medication. No patient changed from HCCP to PGB, whereas many patients treated with PGB changed to HCCP and a few opted to continue with PGB despite its reported efficacy. It is well-admitted that patients prefer topical treatments to oral medications, considering factors such as the onset of effect and the presence or absence of systemic side effects, with local side effects being more acceptable [29]. This finding aligns with those of the ELEVATE study [30]. In this study, the authors demonstrated that treatment with HCCP provided non-inferior pain relief compared to an optimized dose of pregabalin in cases of peripheral neuropathic pain, along with fewer systemic side effects and greater treatment satisfaction, as measured by the Treatment Satisfaction Questionnaire for Medication (TSQM). In our study, 12 out of 25 patients who switched from PGB to HCCP experienced nervous system disorders, which may have influenced their decision to change treatments.

Finally, although quality of life measured by the EQ-5D-5L improved, no difference in EQ-5D-5L scores was observed between groups. This result likely reflects the pain relief observed across all treatment groups and the fact that the EQ-5D-5L, as a generic instrument, may lack sensitivity to detect subtle changes in pain intensity. The limited number of participants and the limited number of participants with clinically relevant anxiety or depressive symptoms (cut-off > 10) at baseline likely limited the ability to detect between-group differences in HADS scores. In Europe, in patients with breast tumors, the prevalence was 38% for anxiety and 27–29% for depression according to the meta-analysis by Martinez-Calderon et al. [31]. Prevalence was 30% and 15% at baseline in our study.

The main strength of the present study is that it is the first to evaluate switching between PGB and HCCP in patients with chronic neuropathic pain treated early after breast surgery. However, the study presents several methodological limitations. As the study started during the COVID-19 pandemic, recruitment was interrupted, resulting in a smaller sample size than initially planned. Moreover, this interruption likely contributed to limiting the number of patients by demotivating some investigators or participants in a context where recruitment is already known to be low [32]. The small and unequal subgroup sizes limited the statistical power for treatment comparisons. To increase the number of participants, particularly in the PGB/HCCP group, we could have combined patients from the PGB/HCCP and PGB + HCCP/HCCP groups (i.e., those who received PGB followed by off-protocol HCCP during the first period of the study and then HCCP during the second period). However, as the protocol was not followed for these patients, their profiles may have differed substantially from those of the other patients, and for greater transparency, we chose to keep the two groups separately. The absence of adjustments for multiple comparisons may also affect the robustness of the reported statistical significance. Nevertheless, the results consistently suggest that HCCP may rapidly alleviate pain or reduce the painful area in some patients with chronic neuropathic pain following breast surgery, whereas others may require continued treatment.

## 5. Conclusions

This first study to examine switching between PGB and HCCP treatments for chronic intercostobrachial neuropathic pain after breast cancer surgery showed patients’ preference for HCCP, confirmed the benefit of repeated HCCP applications, and indicated that HCCP could be preceded by PGB without compromising efficacy or safety. These findings suggest that HCCP may be used early in this population, and PGB initiated before HCCP to relieve patients waiting for HCCP application.

## Figures and Tables

**Table 1:** Patients’ characteristics (*n* = 116).

Characteristics		*N*	%
Age	<65	88	76%
	≥65	28	24%
Professional status	Employed	62	53%
	Retired	40	34%
	Seeking activity	5	4%
	Other	9	8%
Chronic pain syndrome		27	23%
Surgery	Mastectomy	33	28%
	Sentinel lymph node biopsy	72	62%
	Axillary lymph node dissection	40	34%
	No immediate reconstruction	91	78%
Anesthesia	General anesthesia	116	100%
	Paravertebral block	1	1%
	Interpectoral block	3	3%
Postoperative complications	Hematoma	14	12%
	Postoperative infections	2	2%
	Lymphedema	26	22%
Treatment	Adjuvant chemotherapy	35	30%
	Neoadjuvant chemotherapy	23	20%
	Hormonal therapy	81	70%
	Radiotherapy	94	81%
Tumor location	Both	2	2%
	Right	54	47%
	Left	60	52%
Anxiety	Normal	2	2%
	Borderline	40	34%
	Anxiety	31	27%
	MD	29	25%
Depression	Normal	18	16%
	Borderline	66	57%
	Depression	17	15%
	MD	18	16%

MD, missing data. Anxiety and depression were assessed using the Hospital Anxiety and Depression Scale (HADS-A or HADS-D), with 0–7 (normal), 8–10 (borderline), and >10 (anxiety/depression).

**Table 2:** Treatment details.

	Period 1		Period 2		
	HCCP (*N* = 65)	PGB (*N* = 51)	HCCP/HCCP (*N* = 46, 71%)	PGB/HCCP * (*N* = 25, 49%)	PGB/PGB (*N* = 14, 27%)
**HCCP treatment**					
*N* (%):	65 (100%)	-	46/46 (100%)	25/25 (100%)	-
Number of patches, mean (SD):	1.3 (0.7)		1.3 (0.6)	1.2 (0.5)	-
Interval from randomization to administration * (days), median [IQR]:	12 [0–91]		101.5 [63–189]	73 [17–135]	-
**PGB treatment**					
*N* (%):		51 (100%)			14 (100%)
Compliance >75%, *n* (%):		48 (94.1%)			14 (100%)
Maximal dose, mean (SD) - *N* - <100 mg, *n* (%) - 100–150 mg, *n* (%) - >150 mg, *n* (%)		146 (85.5) 50/51 13 (26%) 21 (42%) 16 (32%)			Not recorded
Under treatment (end-of-period)		26 (51%)			14 (100%)

* PGB/HCCP (*N* = 19) or PGB + HCCP/HCCP (*N* = 6). Percentages were calculated based on the total number of patients in the corresponding group. Overall, 19 patients from the HCCP group (HCCP/None) and 12 from the PGB group (PGB/None) had no treatment during Period 2.

## Data Availability

The main data supporting the results of this study are available in this paper. The raw and analyzed datasets generated during this study are available for research purposes from the corresponding author upon reasonable request.

## Supplementary Materials

The following supporting information can be downloaded at: https://www.mdpi.com/article/10.3390/cancers18142355/s1, Table S1: Inclusion and non-inclusion criteria; Table S2: Maximal pain intensity (NRS) from baseline to month 6 by treatment group; Table S3: Painful area (cm^2^) from baseline to month 6 by treatment group; Table S4: Summary statistics of EQ-5D-5L utility score from baseline to month 6 by treatment group (median value); Table S5: Summary statistics of HADS-A and HADS-D scores from baseline to month 6 by treatment group.

## Abbreviations

The following abbreviations are used in this manuscript:

CPBS	chronic pain after breast surgery
CPP	*Comité de Protection des Personnes*
CTCAE	Common Terminology Criteria for Adverse Events
D	Day
DN4	*Douleur Neuropathique en 4 questions*
EQ-5S-L	EuroQol 5-dimension, 5-level questionnaire
HADS	Hospital anxiety-depression scale (HADS-A for anxiety and HADS-D for depression)
HCCP	high-concentration capsaicin (179 mg) patch
IASP	International Association for the Study of Pain
IQR	Interquartile range
M	Month
MD	Missing data
MPI	Maximal pain intensity
NRS	Numeric rating scale
PGB	Pregabalin
SD	Standard deviation
VAS	Visual analogic scale
