# Supplementary material for: High-Concentration Capsaicin Patch and Oral Pregabalin as Second-Line Therapy for Intercostobrachial Neuralgia After Breast Cancer Surgery: Open-Label Follow-Up of a Multicenter Randomized Controlled Clinical Trial
Source: Cancers (Basel). 2026 Jul 21;18(14):2355. doi: 10.3390/cancers18142355 (PMC13406876; doi:10.3390/cancers18142355)
Supplement: Supplementary file 1 [file cancers-18-02355-s001.zip › cancers-4393873-supplementary.pdf]

Table S1. Inclusion and non-inclusion criteria.

Inclusion Criteria	Non-Inclusion Criteria
• Male or female patient.<br>• Age ≥18 years.<br>• Otherwise healthy.<br>• Who has undergone first-intent surgical treatment for breast cancer, regardless of the type of surgery.<br>• With non-irritated skin in the to be treated painful areas.<br>• With confirmed neuropathic pain of the breast and/or axillary area within 3 to 12 months after surgery corresponding to intercostobrachial neuralgia (DN4 score ≥4).<br>• Having signed the written informed consent.<br>• with social security coverage.	• Specific contraindications to one of the two treatments:<br>- Hypersensitivity to PGB or any of its excipients (according to the product information).<br>- Hypersensitivity to HCCP or any of its excipients (according to the product information).<br>• Diabetes.<br>• Prior treatment with HCCP or PGB between surgery and study inclusion.<br>• Current opioid treatment >80 mg/day (oral morphine equivalent) at time of inclusion.<br>• Topical pain treatment administered within 7 days before inclusion.<br>• Uncontrolled hypertension (systolic blood pressure ≥180 mmHg or diastolic blood pressure ≥90 mmHg) or recent cardiovascular events (<3 months) such as stroke, myocardial infarction, pulmonary embolism.<br>• Chronic renal failure.<br>• Pregnant or breastfeeding woman or woman who could become pregnant.<br>• Persons deprived of liberty or under guardianship (including curatorship).<br>• Patient unable to comply with the medical follow-up of the trial.

Table S2. Maximal pain intensity (NRS) from baseline to month 6 by treatment group.

Pain (max, NRS 0-10)	Variable	Total	HCCP /None	HCCP /HCCP	PGB /None	PGB /PGB	PGB /HCCP	PGB+HCCP /HCCP	p-value
D0	N	116	19	46	12	14	19	6	
	Median	6.0	5.0	6.5	6.0	6.0	7.0	6.0	0.5488
	[IQR]	[5.0;7.0]	[4.5;6.0]	[5.0;7.0]	[4.8;8.0]	[4.2;7.8]	[6.0;8.0]	[6.0;6.8]	
M2	N	102	13	41	12	14	18	4	
	Median	5.0	0	5.0	2.5	5.0	6.0	5.0	0.0020
	[IQR]	[2.0;6.0]	[0;4.0]	[4.0;6.0]	[0;4.5]	[2.2;7.0]	[4.2;7.8]	[4.2;6.2]	
M6	N	98	12	41	8	14	18	5	
	Median	4.0	2.0	4.0	3.5	5.5	4.0	5.0	0.4305
	[IQR]	[2.0;6.0]	[0;4.5]	[2.0;6.0]	[2.2;6.2]	[3.0;7.0]	[2.0;4.8]	[5.0;5.0]	
Delta	N	102	13	41	12	14	18	4	
M2-D0	Median	-2.0	-4.0	-1.0	-2.5	-1.5	-1.0	-1.0	0.0169
	[IQR]	[-3.0;0]	[-5.0;-2.0]	[-3.0;0]	[-5.5;-1.8]	[-3.0;-0.2]	[-1.8;0]	[-2.5;0.5]	
Delta	N	98	12	41	8	14	18	5	
M6-D0	Median	-2.0	-3.5	-2.0	-2.5	-1.0	-3.0	-2.0	0.4106
	[IQR]	[-4.0;-0.2]	[-4.2;-1.8]	[-4.0;0]	[-4.2;1.2]	[-2.8;0]	[-4.0;-2.0]	[-2.0;-1.0]	
Delta	N	92	10	38	8	14	18	4	
M6-M2	Median	-0.5	0	-1.0	1.0	0	-2.0	-1.0	0.0103
	[IQR]	[-2.0;1.0]	[0;1.8]	[-2.8;0]	[-2.5;2.2]	[-1.0;1.0]	[-3.0;-1.2]	[-2.5;0]	

Summary statistics are presented as Median [IQR]. P-values were calculated using the non-parametric Kruskal-Wallis test. HCCP, high-concentration capsaicin 179 mg patch; IQR, interquartile; NONE, no treatment; NRS, numeric rating scale (0-10); PGB, PregabalinD0, baseline measurement (day 0); M2, measurement at month 2; M6, measurement at month 6; Delta M2-D0, change from baseline to month 2; Delta M6-D0, change from baseline to month 6; Delta M6-M2, change from month 2 to month 6.

Table S3. Painful area (cm²) from baseline to month 6 by treatment group.

Painful area (surface, cm²)	Variable	Total	HCCP /None	HCCP /HCCP	PGB /None	PGB /PGB	PGB /HCCP	PGB+HCCP /HCCP	p-value
D0	N	114	19	46	11	14	19	5	
	Median	118.9	113.0	118.9	69.3	143.2	127.7	170.3	0.5424
	[IQR]	[55.5;180.4]	[25.0;155.0]	[55.5;184.0]	[42.3;174.8]	[84.7;173.0]	[69.2;193.8]	[90.7;210.5]	
M2	N	106	16	44	9	14	18	5	
	Median	59.7	12.2	67.7	49.3	36.6	132.5	64.1	0.0001
	[IQR]	[31.6;123.5]	[4.6;41.2]	[44.2;105.5]	[19.8;106.3]	[30.5;90.1]	[89.4;165.6]	[36.0;130.5]	
M6	N	100	14	42	7	14	18	5	
	Median	46.7	17.5	55.6	27.6	71.2	47.0	75.3	0.1202
	[IQR]	[16.5;89.1]	[1.0;46.5]	[19.6;88.3]	[1.3;86.4]	[32.6;126.0]	[25.5;81.5]	[20.0;118.7]	
Delta M2-D0	N	105	16	44	8	14	18	5	
	Median	-32.8	-24.9	-40.9	-20.6	-76.0	-11.1	-80.0	0.4577
	[IQR]	[-88.5;4.0]	[-108.1;-1.0]	[-86.2;6.3]	[-44.9;-11.1]	[-118.0;-11.8]	[-63.9;23.0]	[-97.6;-26.6]	
Delta M6-D0	N	99	14	42	6	14	18	5	
	Median	-44.8	-30.3	-44.7	-36.5	-36.7	-84.3	-70.8	0.5291
	[IQR]	[-120.9;-2.7]	[-124.0;-3.9]	[-97.0;11.3]	[-45.4;-25.1]	[-95.5;-5.2]	[-138.3;-25.6]	[-135.2;-26.7]	
Delta M6-M2	N	99	14	42	6	14	18	5	
	Median	-12.7	0	-13.7	-18.0	13.7	-84.8	-44.1	0.0048
	[IQR]	[-57.5;20.1]	[-6.8;18.2]	[-53.9;20.9]	[-31.1;19.7]	[-10.9;41.0]	[-113.0;-35.1]	[-51.3;-16.4]	

Summary statistics are presented as median (IQR). P-values were calculated using the non-parametric Kruskal-Wallis test. HCCP, high-concentration capsaicin 179 mg patch; IQR, interquartile; PGB, pregabalin; SD, standard deviation. D0, baseline measurement (day 0); M2, measurement at 2 months; M6, measurement at 6 months. Delta M2-D0, change from baseline to month 2; Delta M6-D0, change from baseline to month 6; Delta M6-M2, change from month 2 to month 6.

Table S4. Summary statistics of EQ-5D-5L utility score from baseline to month 6 by treatment group (median value).

EQ-5D-5L utility score	Variable	Total	HCCP /None	HCCP /HCCP	PGB /None	PGB /PGB	PGB /HCCP	PGB+HCCP /HCCP	p-value
D0	N	115	19	46	12	14	18	6	
	Median	0.642	0.642	0.732	0.642	0.765	0.642	0.609	0.7277
	[IQR]	[0.487;0.798]	[0.487;0.798]	[0.642;0.798]	[0.490;0.682]	[0.389;0.798]	[0.475;0.798]	[0.380;0.759]	
M2	N	107	16	42	11	14	19	5	
	Median	0.798	0.821	0.798	0.798	0.798	0.732	0.487	0.2988
	[IQR]	[0.642;0.866]	[0.354;0.932]	[0.642;0.798]	[0.687;0.888]	[0.360;0.888]	[0.642;0.798]	[0.487;0.642]	
M6	N	99	15	40	8	14	17	5	
	Median	0.798	0.798	0.732	0.843	0.798	0.798	0.487	0.5305
	[IQR]	[0.642;0.888]	[0.564;0.867]	[0.642;0.821]	[0.709;0.894]	[0.642;0.866]	[0.642;0.798]	[0.254;0.642]	
Delta M2-D0	N	106	16	42	11	14	18	5	
	Median	0	0.101	0	0.155	0.021	0	0	0.6645
	[IQR]	[0;0.156]	[0;0.202]	[0;0.145]	[0;0.274]	[-0.088;0.112]	[0;0.167]	[-0.090;0.090]	
Delta M6-D0	N	99	15	40	8	14	17	5	
	Median	0	0	0	0.122	0	0.112	0	0.4046
	[IQR]	[0;0.156]	[-0.055;0.122]	[-0.010;0.123]	[0;0.218]	[0;0.106]	[0;0.171]	[-0.061;0.090]	
Delta M6-M2	N	97	15	38	8	14	17	5	
	Median	0	-0.090	0	0	0	0	0	0.5532
	[IQR]	[-0.090;0.112]	[-0.112;0.111]	[-0.090;0.090]	[-0.028;0.050]	[-0.090;0.227]	[0;0.156]	[0;0]	

Summary statistics are presented as median (IQR). P-values were calculated using the non-parametric Kruskal-Wallis test. HCCP, high-concentration capsaicin 179 mg patch; IQR, interquartile; PGB, pregabalin D0, baseline measurement (day 0); M2, measurement at 2 months; M6, measurement at 6 months. Delta M2-D0, change from baseline to month 2; Delta M6-D0, change from baseline to month 6; Delta M6-M2, change from month 2 to month 6.

Table S5. Summary statistics of HADS-A and HADS-D scores from baseline to month 6 by treatment group.

HADS Anxiety	Variable	Total	HCCP /None	HCCP /HCCP	PGB /None	PGB /PGB	PGB /HCCP	PGB+HCCP /HCCP	p-value
D0	N	100	16	41	9	13	17	4	
	Median [IQR]	8.0 [6.0;11.0]	8.0 [6.0;11.0]	8.0 [5.0;11.0]	7.0 [6.0;9.0]	9.0 [7.0;11.0]	10.0 [8.0;12.0]	10.5 [8.0;12.5]	0.349
M2	N	85	11	37	6	13	16	2	
	Median [IQR]	8.0 [5.0;10.0]	7.0 [4.0;11.0]	8.0 [5.0;10.0]	4.5 [4.0;5.8]	7.0 [5.0;10.0]	9.5 [7.0;11.0]	8.0 [7.0;9.0]	0.3423
M6	N	88	10	40	6	13	15	4	
	Median [IQR]	8.0 [5.0;11.0]	8.0 [4.0;11.5]	7.0 [5.0;10.2]	4.5 [4.0;10.2]	8.0 [6.0;10.0]	9.0 [7.0;11.0]	7.0 [4.0;10.8]	0.9545
Delta M2-D0	N	85	11	37	6	13	16	2	
	Median [IQR]	0 [-2.0;1.0]	-2.0 [-3.0;0]	0 [-2.0;2.0]	-1.0 [-2.0;1.5]	0 [-2.0;1.0]	0 [-2.0;1.0]	-0.5 [-1.2;0.2]	0.8617
Delta M6-D0	N	83	10	38	5	13	14	3	
	Median [IQR]	-1.0 [-3.0;1.0]	-1.5 [-3.0;0.8]	0 [-2.0;1.0]	-2.0 [-3.0;2.0]	-1.0 [-2.0;2.0]	-1.0 [-3.0;0.8]	0 [-1.5;1.0]	0.9704
Delta M6-M2	N	78	10	36	4	13	13	2	
	Median [IQR]	0 [-1.8;2.0]	0 [-2.5;2.8]	0 [-2.0;2.0]	0 [-1.0;0.5]	1.0 [0;3.0]	-1.0 [-1.0;1.0]	-1.0 [-1.0;-1.0]	0.7449
D0	N	114	18	45	12	14	19	6	0.7696
	Normal	35.96%	38.89%	42.22%	41.67%	28.57%	21.05%	33.33%	
	Borderline	33.33%	27.78%	26.67%	33.33%	42.86%	52.63%	16.67%	
	Anxiety	30.70%	33.33%	31.11%	25.00%	28.57%	26.32%	50.00%	
M2	N	85	11	37	6	13	16	2	0.4905
	Normal	49.41%	63.64%	45.95%	83.33%	53.85%	31.25%	50.00%	
	Borderline	28.24%	9.09%	35.14%	0%	23.08%	37.50%	50.00%	
	Anxiety	22.35%	27.27%	18.92%	16.67%	23.08%	31.25%	0%	
M6	N	88	10	40	6	13	15	4	0.674
	Normal	48.86%	50.00%	55.00%	66.67%	38.46%	33.33%	50.00%	
	Borderline	25.00%	20.00%	20.00%	0%	46.15%	33.33%	25.00%	
	Anxiety	26.14%	30.00%	25.00%	33.33%	15.38%	33.33%	25.00%	
**HADS Depression**	**Variable**	**Total**	**HCCP /None**	**HCCP /HCCP**	**PGB /None**	**PGB /PGB**	**PGB /HCCP**	**PGB+HCCP /HCCP**	**p-value**
D0	N	101	16	42	9	13	17	4	
	Median [IQR]	6.0 [3.0;9.0]	4.0 [3.0;9.2]	6.0 [4.0;9.0]	5.0 [2.0;11.0]	7.0 [3.0;10.0]	6.0 [3.0;8.0]	5.0 [4.2;6.0]	0.9798
M2	N	86	11	37	6	13	16	3	
	Median [IQR]	5.0 [2.0;10.0]	3.0 [1.5;11.5]	5.0 [4.0;10.0]	5.0 [2.0;11.8]	3.0 [2.0;10.0]	5.5 [4.0;9.0]	5.0 [3.5;11.0]	0.7982
M6	N	88	10	40	6	13	15	4	
	Median [IQR]	5.0 [3.0;8.0]	5.5 [3.2;8.0]	5.5 [3.0;9.0]	5.0 [3.0;8.5]	6.0 [2.0;8.0]	4.0 [2.5;7.0]	4.0 [3.0;5.2]	0.7948
Delta M2-D0	N	86	11	37	6	13	16	3	
	Median [IQR]	0 [-2.8;1.0]	0 [-2.5;1.0]	0 [-2.0;1.0]	-1.5 [-3.8;1.5]	-1.0 [-3.0;1.0]	1.0 [-2.0;3.2]	0 [-1.5;4.0]	0.5501
Delta M6-D0	N	84	10	39	5	13	14	3	
	Median [IQR]	-1.0 [-3.0;1.0]	-0.5 [-2.0;0.8]	-1.0 [-2.0;0]	1.0 [-3.0;2.0]	-1.0 [-2.0;1.0]	-1.0 [-3.8;1.8]	-2.0 [-2.5;-1.0]	0.9175
Delta M6-M2	N	79	10	36	4	13	13	3	
	Median [IQR]	0 [-2.0;1.0]	1.0 [-1.0;1.8]	0 [-2.0;1.0]	0.5 [-1.5;1.0]	0 [-1.0;2.0]	-1.0 [-3.0;-1.0]	0 [-5.5;0.5]	0.0798
D0	N	116	19	46	12	14	19	6	0.8941
	Normal	66.38%	63.16%	65.22%	66.67%	64.29%	73.68%	66.67%	
	Borderline	18.10%	21.05%	17.39%	8.33%	14.29%	21.05%	33.33%	

	Depression	15.52%	15.79%	17.39%	25.00%	21.43%	5.26%	0.00%	
M2	N	86	11	37	6	13	16	3	0.7438
	Normal	65.12%	63.64%	67.57%	50.00%	69.23%	62.50%	66.67%	
	Borderline	12.79%	0%	13.51%	16.67%	7.69%	25.00%	0%	
	Depression	22.09%	36.36%	18.92%	33.33%	23.08%	12.50%	33.33%	
M6	N	88	10	40	6	13	15	4	0.8514
	Normal	69.32%	60.00%	67.50%	66.67%	61.54%	80.00%	100.00%	
	Borderline	20.45%	20.00%	20.00%	16.67%	30.77%	20.00%	0%	
	Depression	10.23%	20.00%	12.50%	16.67%	7.69%	0%	0%	

Summary statistics are presented as median (IQR). P-values were calculated using the non-parametric Kruskal-Wallis test. HCCP, high-concentration capsaicin 179 mg patch; IQR, interquartile; PGB, pregabalin. D0, baseline measurement (day 0); M2, measurement at 2 months; M6, measurement at 6 months. Delta M2-D0 change from baseline to month 2; Delta M6-D0, change from baseline to month 6; Delta M6-M2, change from month 2 to month 6.
